# Iron-manganese superoxide dismutases are primarily responsible for antioxidative responses, antibiotic tolerance, and virulence of *Salmonella* Pullorum

**DOI:** 10.1016/j.psj.2026.106638

**Published:** 2026-02-13

**Authors:** Yiluo Cheng, Yanli Li, Rhea Nickerson, Wenting Zhang, Yunqing Guo, Qiao Hu, Qin Lu, Guoyuan Wen, Huabin Shao, Zhenyu Cheng, Qingping Luo, Tengfei Zhang

**Affiliations:** a Key Laboratory of Prevention and Control Agents for Animal Bacteriosis (Ministry of Agriculture and Rural Affairs), Hubei Provincial Key Laboratory of Animal Pathogenic Microbiology, Institute of Animal Husbandry and Veterinary, Hubei Academy of Agricultural Sciences, Wuhan 430064, China; b Department of Microbiology and Immunology, Dalhousie University, Halifax, NS B3H 4H7, Canada; c Hubei Hongshan Laboratory, Wuhan 430064, China

**Keywords:** *Salmonella* Pullorum, Superoxide dismutase, Antioxidative response, Antibiotics tolerance, Virulence

## Abstract

Superoxide dismutase (SOD) is an important component of the bacterial antioxidant system. In *Salmonella* Pullorum, an avian specialist serovar of *Salmonella*, four SODs are encoded, of which *sod1* and *sod3* belong to the Cu/ZnSOD family, and *sod2* and *sod4* belong to the Fe/MnSOD family. However, their respective potential functions in stress responses have remained unclear. In this study, we found that a (p)ppGpp synthetase mutant of *S*. Pullorum exhibited reduced antibiotic tolerance and SOD activity. To further understand the roles of SODs in antibiotic tolerance and virulence, four *sod* single mutants (Δ*sod1,* Δ*sod2,* Δ*sod3*, and Δ*sod4*) and two *sod* double mutants (Δ*sod1*Δ*sod3* and Δ*sod2*Δ*sod4*) were constructed. We found that all four SODs contributed to total SOD activity and superoxide tolerance of *S*. Pullorum. However, this contribution was not uniform, as the Δ*sod2*Δ*sod4* mutant showed the lowest survival rates under paraquat treatment, and the highest reactive oxygen species accumulation and lowest survival rates under antibiotic treatment, suggesting the key roles of *sod2* and *sod4* in degrading ROS in these processes. We also demonstrated that Δ*sod2*Δ*sod4* was more easily eliminated in a chicken macrophage cell, as well as in the spleen and liver of infected chicken hosts, in comparison with the wild-type strain and other *sod* mutants. The Δ*sod2*Δ*sod4* mutant was also the only strain which was completely nonlethal to one-day-old chickens. Taken together, our results indicate that, of the four *sod* genes, the Fe/MnSOD (*sod2* and *sod4*) collectively had the most significant impact on antibiotic tolerance and pathogenicity of *S*. Pullorum.

## Introduction

*Salmonella enterica* is an important zoonotic pathogen worldwide (de Jong, et al., 2012). *Salmonella enterica* serovar Pullorum (*S*. Pullorum) has strict host specificity for poultry (Zhou, et al., 2023), and can cause severe infectious pullorum disease (PD) in chickens, making it a serious threat to the poultry industry (Guo, et al., 2018). The widespread use of antibiotics lead to a growing number of multidrug-resistant *S*. Pullorum (Li, et al., 2022). In order to be successful during infection, *Salmonella* requires strategies to survive host immune defenses, as well as antibiotic treatment. A better understanding of *Salmonella*’s mechanisms to resist these stresses will help us develop strategies to control this pathogen better.

Oxidative metabolism is an important link in the process of converting nutrients into energy, and endogenous reactive oxygen species (ROS) are mainly produced by autoxidation and electron divulge that occur in metabolic processes (Meylan, et al., 2017). ROS mainly include superoxide anions (O_2_^-^), hydroxyl radicals (OH^.-^), and hydrogen peroxide (H_2_O_2_) (Dryden, 2018). Under normal conditions, the bacterial oxidative stress response can balance ROS produced during bacterial metabolism. However, antibiotic treatment can cause serious metabolic disturbance, resulting in excessive intracellular accumulation of toxic ROS (Brynildsen, et al., 2013). An overaccumulation of ROS damages biological macromolecules in cells, causing DNA breakage, protein carbonylation, and lipid peroxidation, amongst other insults, which leads to the death of bacteria (Goswami, et al., 2006). Therefore, in addition to the direct antibiotic attack to the targets, intracellular ROS accumulation is an important factor which enhances the lethality of antibiotics (Dryden, et al., 2017). Additionally, the phagocytic oxidative burst is a primary effector of innate immunity that protects against bacterial infection (Cavinato, et al., 2020). When pathogens invade the body, phagocytic cells represented primarily by macrophages and neutrophils, immediately respond, recognize and phagocytose pathogens, and mobilize NADPH oxidase and mitochondria to release a large quantity of ROS into phagocytic vesicles to achieve the killing and elimination of phagocytosed pathogens (Roca, et al., 2022).

In order to cope with oxidative stress and protect themselves from the damaging effects of ROS, bacteria can activate a complex multi-level antioxidant system involving enzymes such as superoxide dismutase (SOD), catalase, peroxide reductase, and hydrogen peroxide tolerance protein, which maintain ROS in the bacterial cell at a dynamic balance under normal conditions (Zhang, et al., 2012). SODs specifically catalyze the dismutation of the deleterious superoxide anion (O_2_-) to form hydrogen peroxide (H_2_O_2_) and oxygen (O_2_). H_2_O_2_ is then in turn metabolized by catalases (Gao, et al., 2019). SODs are metalloenzymes, and in most bacteria they are classified into two main families depending on their metal cofactor: Cu/ZnSOD or Fe/MnSOD. The latter family can be further divided into FeSOD and MnSOD subtypes (Tidhar, et al., 2015). Different bacterial species have varying numbers of SODs belonging to different families. For example, *Escherichia coli* encodes three SODs belonging to Cu/ZnSOD or Fe/MnSOD families (Gao, Xia, Wang, Ye, Liu and Gao, 2019), but most *Listeria monocytogenes* and *Pseudomonas aeruginosa* strains encode only Fe/MnSOD family SODs (Hassett, et al., 1995). In addition, nickel SOD has been found in some species, such as *Streptomyces* (Kim, et al., 2014). All *Salmonella* encode several SOD proteins in their genomes, such as *S.* Pullorum, which encodes 4 SOD proteins. Some studies have investigated the role of one SOD at a time in some serotypes of *Salmonella,* but have not comprehensively examined the roles of multiple SODs in a holistic manner (Tidhar, Rushing, Kim and Slauch, 2015; Wang, et al., 2019). In our previous study on the (p)ppGpp synthetase of *S.* Pullorum, we found that knockout of (p)ppGpp synthetase in *S.* Pullorum (Δ*relA*Δ*spoT*) led to reduced minimal bactericidal concentrations (MBC) of ciprofloxacin and ampicillin, as well as pathogenicity. Interestingly, RNA sequencing revealed that *sod* genes were downregulated in (p)ppGpp synthetase mutant Δ*relA*Δ*spoT* (Wang, et al., 2021). In *P. aeruginosa*, SOD activity has been found to be important on its tolerance to ofloxacin and gentamicin (Martins, et al., 2018). However, whether SODs play important roles in antibiotic resistance and virulence, and the respective role of *S*. Pullorum’s four SODs in these phenotypes, remained unknown.

In this study, four *sod* single mutants, and two *sod* double mutants (knockout of both *sod* genes belonging to the same metal cofactor family) of *S.* Pullorum were constructed, and their antioxidation function and antibiotic resistance abilities, as well as their virulence, were investigated. This study will help us to further understand the contribution of SODs to antibiotic tolerance and pathogenesis in *Salmonella*.

## Materials and methods

### Bacterial strains, plasmids and growth conditions

The strains used in this study are listed in Table 1. *S*. Pullorum C79-3, which is a virulent strain, was obtained from the China Institute of Veterinary Drug Control, and stored at our lab. The Δ*relA*Δ*spoT* mutant of C79-3 was constructed in our previous study, and stored at our lab (Wang, Cheng, Zhang, Lu, Wen, Luo, Shao, Pan and Zhang, 2021). All the *S*. Pullorum strains, including the C79-3 wild-type strain and its derivatives were grown at 37°C in LB broth.

**Table 1:** Bacterial strains, cells and plasmids used in this study.

Strain, plasmid, or cell line		Characteristics	Preservation	Source or reference
Strains				
C79-3		Wild-type virulent *S*. Pullorum strain	Stored at −80 °C in 20% (v/v) glycerol	CVCC519 (Wang, Cheng, Zhang, Lu, Wen, Luo, Shao, Pan and Zhang, 2021)
Δ*relA*Δ*spoT*		*relA* and *spoT* double deletion mutant of C79-3		(Wang, Cheng, Zhang, Lu, Wen, Luo, Shao, Pan and Zhang, 2021)
Δ*sod1*		*sod1* deletion mutant of C79-3		In this study
Δ*sod2*		*sod2* deletion mutant of C79-3		In this study
Δ*sod3*		*sod3* deletion mutant of C79-3		In this study
Δ*sod4*		*sod4* deletion mutant of C79-3		In this study
Δ*sod1*Δ*sod3*		*sod1* and *sod3* double deletion mutant of C79-3		In this study
Δ*sod2*Δ*sod4*		*sod2* and *sod4* double deletion mutant of C79-3		In this study
Plasmids				
pKD3		Carry *cat* gene, template plasmid	Stored at −20 °C; keeped in *E*. coli DH5α	(Datsenko and Wanner, 2000)
pKD46		Amp^r^; expresses λRed recombinase		(Datsenko and Wanner, 2000)
pCP20		Amp^r^Cm^r^, Expresses FLP recombinase,		(Datsenko and Wanner, 2000)
pUC19		Cloning vectors, Amp^r^		Purchased from NEB (N3041S)
Cells				
Cell line HD11		Chicken macrophage line	Stored in liquid nitrogen	(Gao, Xia, Wang, Ye, Liu and Gao, 2019)

The λRed system was used for construction of the *sod* mutants as previously described (Datsenko and Wanner, 2000). The primers used are listed in Table S1. As an example, to knockout *sod1*, the chloramphenicol resistance cassette (*cat*) was amplified from the plasmid pKD3 by PCR using primers R-sod1-F and R-sod1-R, which contained the sequences of homologous recombination arms of *sod1*. The PCR parameters were set as 95°C for 5 min, followed by 35 cycles of 95°C for 15 s, 60°C for 15 s and 72°C for 60 s. The amplified *cat* was transformed into C79-3 strain containing plasmid pKD46. The *sod1* gene was replaced with the *cat* gene, and then the *cat* gene was removed using the plasmid pCP20, generating the Δ*sod1* mutant. Using the same method, Δ*sod2*, Δ*sod3*, and Δ*sod4* single mutants, and Δ*sod1*Δ*sod3* and Δ*sod2*Δ*sod4* double mutants were constructed. All the mutants were confirmed by PCR and DNA sequencing. To produce the complementary strain, the plasmid pUC19 carrying the *sod* cassette was electro-transformed into the mutant, and expression was confirmed by RT-PCR. All the strains were added with a final concentration of 25% glycerol and were stored at −80°C.

### Antibiotic tolerance assays

To compare the antibiotic tolerance of different mutants, the survival curves of strains after antibiotics treatment were measured. Briefly, the bacteria were suspended in PBS and diluted to approximate 10^7^ CFU/mL, and then exposed to LB broth containing 128 µg/mL ampicillin (1 MBC) or 0.5 µg/mL ciprofloxacin (0.5 MBC) at 37°C with shaking at 200 rpm. To evaluate the effect of antioxidant treatment on the antibiotic killing, 10 mM GSH was added to the LB medium as previously reported (Goswami, Mangoli and Jawali, 2006). Following treatment with antibiotics for 2, 4, 6 and 8 h, the bacteria were serially diluted and plated on LB plates to determine the remaining CFU. All tests were performed in triplicate, and data represent the averages of three independent assays.

### Total SOD activity assays

The WST-8 method was used to detect the total SOD activity of each strain (Yang, et al., 2020). WST-8 reacts with O_2_- catalyzed by xanthine oxidase to produce formazan dye, while SOD can catalyze the dismutation of O_2_-, so this step can be inhibited by SOD. Therefore, the activity of SODs is negatively correlated with the production of formazan dye. In this study, overnight cultured strains were diluted 1:100 in fresh LB medium, and then grown to logarithmic phase (OD_600_ = 0.5). The strains were collected and washed with PBS. The SOD activities in the samples were measured using Total Superoxide Dismutase Assay Kit with WST-8 (Beyotime, China). In brief, the collected samples were lysed using SOD sample preparation buffer, followed by centrifugation at 12,000 × *g* for 5 minutes. The SOD activities of the resulting supernatants were tested according to the manufacturer’s instruction. The absorbance of the sample was measured at 450 nm, and calculated as the inhibition rates of the formazan dye production after addition of the bacteria samples. To test the total SOD activity after antibiotic treatment, ciprofloxacin (final concentration = 0.15 µg/mL) was added, and the samples were collected after ciprofloxacin treatment for 10 mins.

### Real-time reverse transcription-PCR

Real-time reverse transcription PCR (RT-qPCR) was carried out to test the expression of *sod* genes in Δ*relA*Δ*spoT* and *sod* mutants using SYBR Green Master Mix (Vazyme, Nanjing, China). The primers are provided in Table S1. Overnight cultured strains were 1:100 diluted in fresh LB medium, and grown to logarithmic phase (OD_600_ = 0.5), and then 2 ml of samples were collected for testing. To detect the effect of antibiotic treatment on the expression of *sod* genes, ciprofloxacin (final concentration = 0.15 µg/mL) was added, and the samples were collected after ciprofloxacin treatment for 10 mins. Following total RNA isolation and reverse transcription, the expression of each gene of interest was tested by qPCR and calculated using the 2^-ΔΔCt^ method. The data was normalized to the endogenous control *gapdh*. All tests were performed with three independent replicates.

### Superoxide tolerance assays

Superoxide tolerance was evaluated by measuring the survival rates of bacteria after paraquat treatment (Sigma-Aldrich, USA). Briefly, overnight cultured strains were diluted 1:100 in fresh LB medium, then the bacteria were suspended in PBS and diluted to 10^6^ CFU/mL, and then exposed to LB broth containing 30 μg/mL paraquat at 37°C. Following 1 h of paraquat exposure, bacteria were serially diluted and plated on LB plates. Survival rate was calculated as the number of bacteria remaining after exposure divided by the initial number of bacteria. All tests were performed in triplicate, and data represent the averages of three independent assays.

### ROS measurement

ROS were measured using the probe DCFH-DA according to the instructions of the Fluorometric Intracellular ROS Assay Kit (Beyotime, China) (Xia, et al., 2021). Briefly, overnight cultured strains were diluted 1:100 in fresh LB medium, then the bacteria in exponential growth period (OD_600_ = 0.5) were washed twice and resuspended in 20 mL PBS. To test the induction of ROS, ciprofloxacin (final concentration = 0.125 µg/mL) was added, and 2 mL samples were collected every five mins. The ROS was labeled with a DCFH-DA fluorescent probe at a final concentration of 1 × and incubated for 1 h according to the manufacturer’s instructions, and fluorescence intensity was measured using a microplate reader (λ_ex_=490/λ_em_=525 nm).

### Detection of the oxidative damage to biological macromolecules

Protein carbonyl is an early marker in the process of protein molecules being modified by free radical oxidation, and its content indicates the degree of oxidative damage of protein (Georgiou, et al., 2018). Lipid Peroxide (LPO) is the peroxide produced after the action of reactive oxygen species on unsaturated fatty acid chains, and its content is the main index of the degree of lipid oxidative damage (Lund, et al., 2007). To test the oxidative damage to protein and lipid during antibiotic treatment, 1 mL of cells were collected after 0.125 µg/mL ciprofloxacin treatment. Then, the collected cells were washed with PBS and disrupted using ultrasonic (5-10 seconds of sonication followed by 20-30 seconds of rest, 2 mins). The protein carbonyl content of the disrupted cells was determined by 2, 4-dinitrophenylhydrazine (DNPH) colorimetry using Protein Carbonyl Content Assay kit (Solarbio, Beijing, China) according to manufacturer’s instructions. The LPO content of the disrupted cells was detected using LPO Content Assay kit (Solarbio, Beijing, China) according to instructions. To evaluate the effect of antioxidant on oxidative damage, a final concentration of 10 mM GSH was added to the medium at the beginning of the culture.

### Ingestion and intracellular survival assays

Internalization and proliferation of bacteria in the chicken macrophage cell line HD11 was carried out as previously described (Lissner, et al., 1983). The HD11 cell line was cultured in RPMI1640 (Hyclone, USA) with 10% fetal bovine serum (PAA, Pasching, Australia), and maintained at 37°C in a 5% CO_2_ environment with 5 × 10^5^ cells per well in 24-well cell culture plates. Bacteria were added to the cells (MOI = 100) and 1 h was allowed for ingestion. The wells were washed with PBS, and the extracellular bacteria were killed by treatment with 50 μg/mL gentamycin. At different incubation periods (0, 3, 7 and 10 h), the macrophages were washed with PBS and lysed with 1% Triton X-100 (Ameresco, USA). The lysates were plated onto LB plates for determining CFU of intracellular bacteria. Intracellular survival and growth were determined as the fold changes in the CFU at different incubation time by comparing with the 0 h. The assays were carried out for three biological replicates.

### Colonization and virulence assays

The pathogenicity of the *sod* mutants was determined using a specific pathogen-free (SPF) chicken infection model. Eighty one-day-old chickens were randomly assigned into eight groups (*n* = 10) according to ARRIVE guidelines. Chickens were intraperitoneally inoculated with approximately 1.5 × 10⁸ CFU/chicken of wild-type strain or different *sod* mutants, respectively, and PBS was injected as a negative control. To minimize observer bias, investigators responsible for inoculation, daily survival monitoring, and data recording were blinded to group allocation throughout the experiment. The survival of all chickens was recorded for 14 consecutive days, and the remaining chickens were euthanized with an intramuscular injection of sodium pentobarbital (100 mg/kg bodyweight) at the end of the study.

To compare the colonization ability of each strain, 96 chickens were randomly divided into eight groups and injected intramuscularly with 1 × 10^7^ CFU/chicken of each strain, respectively. Three chickens within each group were sacrificed 1-, 4-, 7- and 14-days post-infection. Then, 0.25 g liver or spleen were collected, and triturated in 1 ml PBS, and the viable numbers of bacteria in the liver and spleen were counted by CFU plating on LB plates.

### Statistical analysis

All experimental data are presented as means ± standard deviation and were analyzed using GraphPad INSTAT (GraphPad Software, Inc., San Diego, CA, USA). Statistical significance was assessed using unpaired Student’s t-tests for pairwise comparisons and one-way analysis of variance for multiple-group comparison. Statistical significance was defined as a *P* value <0.05.

## Results

### The antibiotic tolerance of the (p)ppGpp synthetase mutant ΔrelAΔspoT was reduced in S. Pullorum

**Fig. 1:**
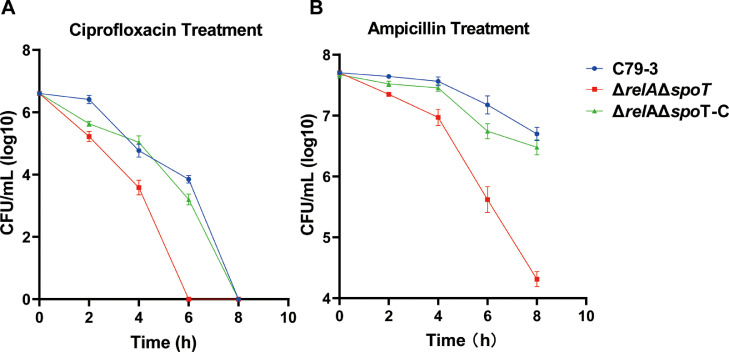
The time-killing curves after antibiotics treatment. Wild-type strain C79-3, Δ*relA*Δ*spoT* mutant and its complementary strain (10^7^ CFU/mL) were exposed to LB broth containing 0.5 µg/mL ciprofloxacin (A) or 128 µg/mL ampicillin (B). Following treatment with antibiotic for 2, 4, 6 and 8 h, the bacteria were serially diluted and plated on LB plates to determine the remaining CFU. Data represent the mean ± standard deviation of three independent replicates. The CFU of Δ*relA*Δ*spoT* mutant and its complementary strain at each time point were compared to the wild-type strain using an unpaired t-test. *P* < 0.05 indicates a statistically significant difference compared to the wild-type.

In our previous study, we found that the MBCs to ciprofloxacin and ampicillin were reduced more than four-fold in the (p)ppGpp synthetase mutant (Δ*relA*Δ*spoT*) of *S*. Pullorum compared to the wild-type strain C79-3 (Wang, Cheng, Zhang, Lu, Wen, Luo, Shao, Pan and Zhang, 2021). To further confirm the role of (p)ppGpp synthetase in antibiotic tolerance, we compared the time-killing curves of Δ*relA*Δ*spoT* with C79-3. As shown in Fig. 1A, the Δ*relA*Δ*spoT* mutant showed significantly reduced survival rates by comparing with C79-3 under ciprofloxacin at 2 and 4 hours after treatment (*P*<0.05), and was completely eliminated 6 hours after treatment. On the other hand, more than 10^3^ CFU/mL of the C79-3 and the complemented strain were still alive 6 hours after treatment and they were only eradicated 8 hours after treatment. The Δ*relA*Δ*spoT* mutant also showed a remarkably reduced survival rate under ampicillin treatment (Fig. 1B).

### The total SOD activity of S. Pullorum was reduced in the (p)ppGpp synthetase mutant

**Fig. 2:**
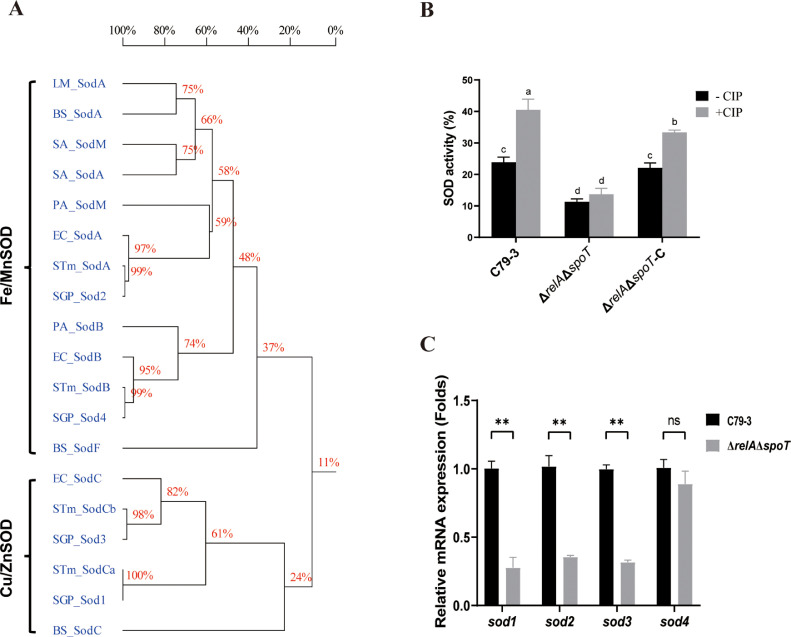
The total SOD activity and the expression levels of *sod* genes in the Δ*relA*Δ*spoT* mutant compared to wild-type strain C79-3. (A) A homology tree of SOD proteins from selected bacteria was constructed based on a multiple sequence alignment using DNAMAN. The reference strains include *L. monocytogenes* ATCC 19117 (LM), *P. aeruginosa* PAO1 (PA), *E. coli* K-12 MG1655 (EC), *S. aureus* MRSA252 (SA), *B. subtilis* 168 (BS), *S*. Typhimurium SL1344 (STm), *S*. Pullorum RKS5078 (SGP). (B) The total SOD activity of Δ*relA*Δ*spoT* and its parent strain (C79-3) and complementary strain (Δ*relA*Δ*spoT*-C). The total SOD activity was detected before (-CIP) and after (+CIP) ciprofloxacin (CIP) treatment for 10 mins. Data were analyzed by one-way ANOVA with Dunnett’s *post-hoc* test. Bars sharing no common letter (a, b, c, d) are significantly different (*P* < 0.05). (C) Relative expression of *sod* genes in the Δ*relA*Δ*spoT* mutant relative to strain C79-3 after 10 min of ciprofloxacin treatment. Statistical significance was determined by an unpaired t-test, ** *P* < 0.01.

SODs were previously reported to be important for antibiotic tolerance in *P. aeruginosa* (Martins, McKay, Sampathkumar, Khakimova, English and Nguyen, 2018). As shown in Fig. 2A, 4 SODs are encoded in the genome of *S.* Pullorum. Genes *sod1* (SPUL_1714) and *sod3* (SPUL_1255) encode Cu/ZnSOD respectively, which contains a Sod_Cu motif, and the proteins showed high similarity to Cu/ZnSODs in *E. coli* and *Salmonella* Typhimurium and *Bacillus subtilis*. Genes *sod2* (SPUL_3610) and *sod4* (SPUL_1245) encode Fe/MnSOD respectively, which contains a Sod_Fe_N motif and a Sod_Fe_C motif both, and the proteins showed high similarity to Fe/MnSODs in *L. monocytogenes, B. subtilis, Staphylococcus aureus, P. aeruginosa, E. coli* and *S.* Typhimurium.

To understand the link between SODs and (p)ppGpp synthetase in *S*. Pullorum, we compared the total SOD activity of Δ*relA*Δ*spoT* with C79-3. As shown in Fig. 2B, the total SOD activity of C79-3 was significantly induced after ciprofloxacin treatment for 10 mins. In contrast, the total SOD activity of Δ*relA*Δ*spoT* was approximately two-fold lower than C79-3 in the absence of antibiotic, and could not be induced by ciprofloxacin treatment (Fig. 2B). As showed in Fig. 2C, we then quantified the expression of *sod* genes, and found that the expression of *sod1, sod2* and *sod3* genes were decreased approximately three-fold in Δ*relA*Δ*spoT* compared to C79-3 under ciprofloxacin treatment. These results suggest that the total SOD activity of *S*. Pullorum is RelA/SpoT-dependent.

### The effect of sod deletion on superoxide tolerance

**Fig. 3:**
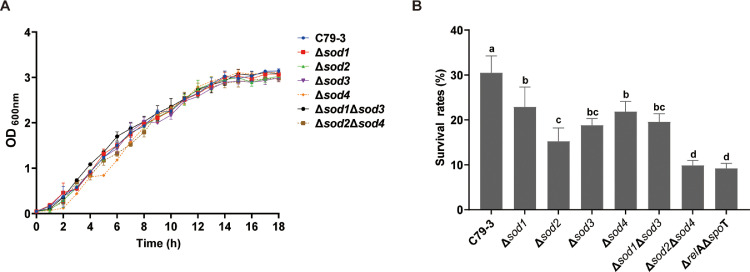
The growth curves and survival rates. (A) The growth curves of four *sod* single mutants (Δ*sod1*, Δ*sod2*, Δ*sod3,* and Δ*sod4*), and two *sod* double mutants (Δ*sod1*Δ*sod3* and Δ*sod2*Δ*sod4*) versus the wild-type C79-3 strain. Data represent the mean ± standard deviation of three independent replicates. (B) Comparison of the survival rates of *sod* mutants and Δ*relA*Δ*spoT* mutant after 30 μg/ml paraquat treatment for one hour. The data are the means and standard deviations of three independent experiments. Statistical significance was determined by one-way ANOVA with Tukey’s honestly significant difference (HSD) *post-hoc* test. Bars sharing no common letter (a, b, c, d,) are significantly different (*P* < 0.05).

In order to investigate the contribution of each SOD to oxidative stress tolerance, four single *sod* mutants (Δ*sod1*, Δ*sod2*, Δ*sod3,* and Δ*sod4*) and two double mutants (Δ*sod1*Δ*sod3* (Cu/ZnSODs) and Δ*sod2*Δ*sod4* (Fe/MnSODs)) were constructed. These mutants showed no significant differences in growth rates or OD_600_ values relative to the wild-type strain (mean ± SD, *n* = 3; *P* > 0.05; Fig. 3A). We also tested the expression of *sod* genes in each *sod* mutant, and found that knockout of one *sod* gene did not change the expression level of any of the other three *sod* genes (data not shown).

As SODs are components of the oxidative stress response system, we first compared the ability of these *sod* mutants to tolerate paraquat, which was the superoxide generators (details were in table S2). As shown in Fig. 3B, the survival rates of all the mutants were significantly (*P* < 0.01) reduced compared to C79-3 after paraquat treatment. Among these *sod* mutants, Δ*sod2*Δ*sod4* showed the lowest survival rate (11.7%), similar to Δ*relA*Δ*spoT* (11.2%). In contrast, the survival rates of other *sod* mutants, including the four *sod* single mutants and the Δ*sod1*Δ*sod3* double mutant, were lower than C79-3 (30.3% survival), but significantly (*P* < 0.01) higher than Δ*sod2*Δ*sod4*, ranging from 23.8% to 16.4%. These results suggest that all four SODs contribute to superoxide tolerance; however, the Fe/MnSOD family (*sod2* and *sod4*) collectively had the most significant impact on the superoxide tolerance of *S*. Pullorum.

### The role of SODs in antibiotic tolerance

**Fig. 4:**
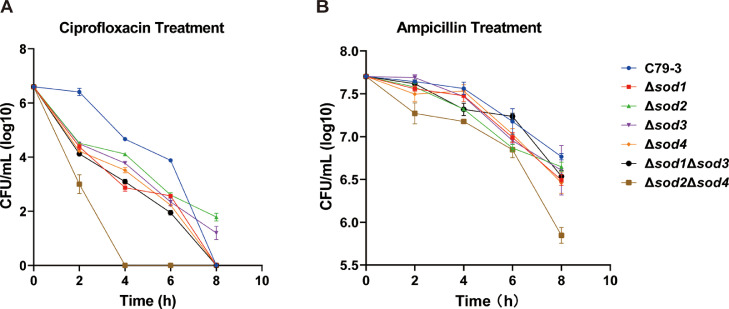
Comparison of the antibiotic tolerance of four *sod* single mutants (Δ*sod1*, Δ*sod2*, Δ*sod3,* and Δ*sod4*) and two *sod* double mutants (Δ*sod1*Δ*sod3* and Δ*sod2*Δ*sod4*) of *S*. Pullorum compared to C79-3. (A) The time-killing curves during ciprofloxacin treatment. (B) The time-killing curves during ampicillin treatment. Data represent the mean ± standard deviation of three independent replicates. The CFUs of each mutant at each time point was compared to the wild-type strain using an unpaired t-test. *P* < 0.05 indicates a statistically significant difference compared to the wild-type.

To further reveal the role of SODs in promoting *S.* Pullorum antibiotic tolerance, the survival of these *sod* mutants after antibiotic treatment was compared with C79-3. Ampicillin and ciprofloxacin, which target the cell wall and DNA replication respectively, were chosen because of their differing mechanisms of action and clinical relevance. As shown in Fig. 4, the survival of Δ*sod2*Δ*sod4* was dramatically reduced with either ampicillin or ciprofloxacin treatment. In the ciprofloxacin treatment group, the Δ*sod2*Δ*sod4* mutant had the fewest surviving bacteria at two hours post treatment by comparing with other groups (*P* < 0.05), and then was nearly eliminated at four hours post treatment, while the other mutants still persisted with more than 10^2^ CFU/mL bacteria at this point (Fig. 4A). In the ampicillin treatment group, the Δ*sod2*Δ*sod4* also had the lowest survival rates compared to C79-3 and the other *sod* mutants after 8 hours of treatment (*P* < 0.05) (Fig. 4B). These results indicate that the Fe/MnSOD family (*sod2* and *sod4*) collectively play a major role in the antibiotic tolerance of *S*. Pullorum.

### ROS accumulation and total SOD activity of sod mutants after antibiotic treatment

**Fig. 5:**
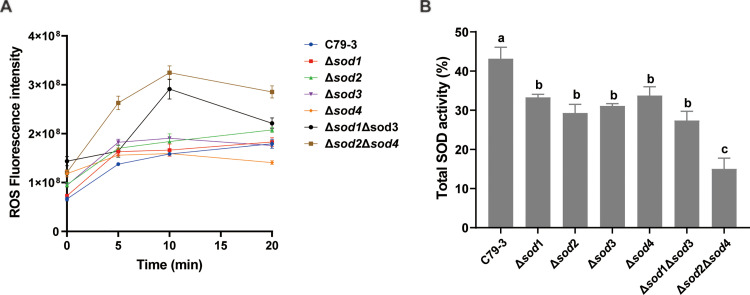
The ROS accumulation and total SOD activity of four *sod* single mutants (Δ*sod1*, Δ*sod2*, Δ*sod3,* and Δ*sod4*) and two *sod* double mutants (Δ*sod1*Δ*sod3* and Δ*sod2*Δ*sod4*) after ciprofloxacin treatment. (A) The ROS accumulation of each mutant at 5-, 10- and 20-min post ciprofloxacin treatment. (B) The total SOD activity of each mutant after ciprofloxacin treatment for 10 mins. Statistical significance was determined by one-way ANOVA with Dunnett’s *post-hoc* test. Bars sharing no common letter (a, b, c) are significantly different (*P* < 0.05).

Endogenous toxic ROS produced by metabolic disturbance during antibiotic treatment is very harmful to bacteria (Brynildsen, Winkler, Spina, MacDonald and Collins, 2013). Therefore, the ability of bacteria to degrade excessive ROS is important for their antibiotic tolerance. We compared the accumulation of ROS in *sod* mutants using ciprofloxacin treatment. As shown in Fig. 5A, ciprofloxacin treatment rapidly induces the accumulation of ROS in all strains, with the greatest accumulation occurring in the Δ*sod2*Δ*sod4* mutant at 5-, 10- and 20-mins post ciprofloxacin treatment compared to other strains (*P* < 0.05). This suggests that knockout of *sod2* and *sod4* collectively greatly abrogated the ability of *S.* Pullorum to degrade ROS induced by antibiotic treatment. The Δ*sod1*Δ*sod3* mutant at 10- and 20-mins post ciprofloxacin treatment also accumulated higher amounts of ROS amount compared to C79-3 (*P* < 0.05). However, the single mutants did not accumulate significantly more ROS amounts than C79-3.

Above, we found that total SOD activity could be induced by ciprofloxacin treatment (Fig. 2B). To understand whether the difference in ROS accumulation of each mutant during ciprofloxacin treatment was due to differences in their total SOD activity, we compared their total SOD activity after ciprofloxacin treatment. As shown in Fig. 5B, the total SOD activity of all the mutants were reduced compared to C79-3 (*P* < 0.05), and the double mutants, especially Δ*sod2*Δ*sod4*, had lower SOD activity than all of the single mutants (*P* < 0.01). The total SOD activity of these mutants negatively correlates with their accumulation of ROS, suggesting a major role of SODs in restricting harmful ROS within *S.* Pullorum.

### The antioxidant glutathione alleviated ROS damage in sod mutants during ciprofloxacin treatment

**Fig. 6:**
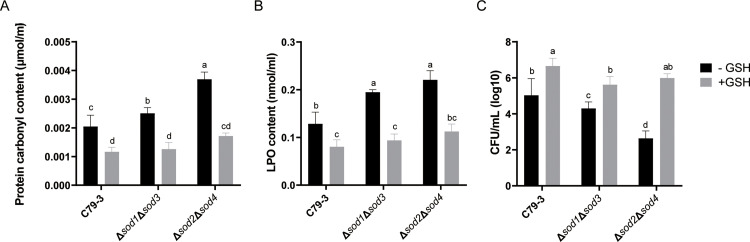
Comparison of ROS damage and survival rates between C79-3 and two *sod* double mutants (Δ*sod1*Δ*sod3* and Δ*sod2*Δ*sod4*) after ciprofloxacin and/or GSH treatment. (A) Protein carbonyl content, which indicates oxidative damage to protein in each mutant after ciprofloxacin treatment. (B) Lipid peroxide (LPO) content, which indicates lipid oxidative damage in each mutant after ciprofloxacin treatment. (C) The number of surviving bacteria quantified in CFU/mL after ciprofloxacin treatment for two hours. “+GSH” means the reducing agent GSH was added in the cultures. “-GSH” means GSH was not added. Statistical significance was determined by one-way ANOVA with Dunnett’s *post-hoc* test. Bars sharing no common letter (a, b, c, d) are significantly different (*P* < 0.05).

As above described, *sod2* and *sod4* collectively played the major roles in antibiotic tolerance. To further confirm these roles, we quantified the levels of the oxidative damage to proteins and lipids in Δ*sod2*Δ*sod4* mutant bacteria during ciprofloxacin treatment, and evaluated the protective effect of treating bacteria with the reducing agent glutathione (GSH) during this stress. Δ*sod1*Δ*sod3* and the wild-type C79-3 were also compared. As shown in Fig. 6A and 6B, the Δ*sod2*Δ*sod4* mutant had the highest protein carbonyl content and lipid peroxide (LPO) content, which suggested that these bacteria suffered severe oxidative damage during ciprofloxacin treatment. Compared with C79-3, Δ*sod1*Δ*sod3* also contained more LPO content. At the same time, addition of GSH could reduce the protein carbonyl content and LPO content significantly in all of the strains. This effect was most significant for Δ*sod2*Δ*sod4*. Even with the addition of GSH, the protein carbonyl content and LPO content were still higher in Δ*sod2*Δ*sod4* than other two strains, although this difference was not significant. When GSH was added, each strain also showed increased survival following ciprofloxacin treatment, with the Δ*sod2*Δ*sod4* mutant now surviving comparably to C79-3 (Fig. 6C). These results further indicate that Fe/MnSOD (*sod2* and *sod4*) play a crucial antioxidative function which allows *S*. Pullorum to resist antibiotic-mediated oxidative damage.

### The effect of SODs on survival and proliferation of S. Pullorum in chicken macrophages

**Fig. 7:**
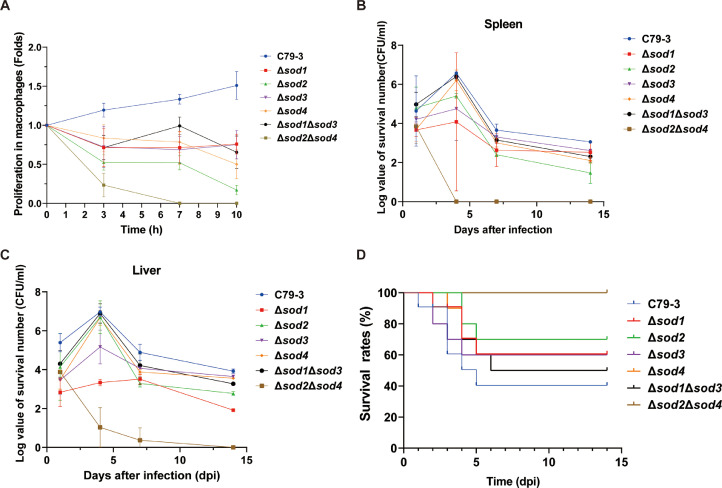
Evaluation of the effect of *sod* on colonization and virulence (A) The intracellular proliferation of Δ*sod1*, Δ*sod2*, Δ*sod3,* and Δ*sod4* single mutants, and Δ*sod1*Δ*sod3* and Δ*sod2*Δ*sod4* double mutants in chicken macrophage HD-11 cells. (B) The colonization of *sod* mutants in spleens of chickens. (C) The colonization of *sod* mutants in livers of chickens. (D) The survival rate of chickens infected with *sod* mutants. Data represent the mean ± standard deviation of three independent replicates. The area under the curve (AUC) was determined for each curve and compared to the wild-type C79-3 using an unpaired t-test with Welch’s correction. *P < 0.05 indicates a statistically significant difference compared to the wild-type.

Generation of ROS in the form of oxidative burst is one of the most important mechanisms by which macrophages kill phagocytosed pathogens (Cavinato, Genise, Luly, Di Domenico, Del Porto and Ascenzioni, 2020). Therefore, to determine whether *sod* genes contribute to the survival of *S*. Pullorum within macrophages, we compared the proliferation of *sod* mutants with C79-3 in HD11 chicken macrophage cells. As shown in Fig. 7A, only C79-3 was able to proliferate well in HD11 cells, while all of the mutants had impaired proliferation to varying degrees. As expected based on previous results, the Δ*sod2*Δ*sod4* mutant was least able to persist in macrophages, and was nearly eliminated by 7 hours post infection, whereas the other mutants persisted until the endpoint of 10 hours post-infection.

### Colonization and virulence of sod mutants in chicken

To evaluate the role of SODs in the colonization ability of *S*. Pullorum, one-day-old chickens were inoculated with 1 × 10^7^ CFU/chicken of C79-3 or *sod* mutants. As shown in Fig. 7B and C, the Δ*sod2*Δ*sod4* mutant was least able to persist in the chicken host, as it was almost eliminated at 4 days post infection (dpi) in the spleen and at 14 dpi in the liver. By contrast, other strains were briefly able to proliferate before 4 dpi, after which their bacterial loads were reduced but still able to persist in the chicken at 14 dpi.

We then evaluated the effect of SODs on the pathogenicity of *S*. Pullorum. One-day-old chickens were inoculated with 1.5 × 10^8^ CFU/chicken of each strain. As shown in Fig. 7D, this dose resulted in a survival rate of 40% (4/10) in the wild-type C79-3 infection group. However, 100% of the chickens in the Δ*sod2*Δ*sod4* infection group survived. The groups infected with other mutants also had increased survival rates compared to the C79-3 infection group, ranging from 50% to 70%. These results suggest that SODs contribute to the pathogenicity of *S*. Pullorum, and among them, Fe/MnSOD (*sod2* and *sod4*) collectively, are required for the effective colonization and pathogenicity of *S*. Pullorum in chickens.

## Discussion

As a pathogen, *Salmonella* must overcome diverse stress conditions in order to survive during infection. Antibiotic treatment and the host immune response are two of the most severe challenges to the survival of pathogens during infection (de Jong, Parry, van der Poll and Wiersinga, 2012). During these two challenges, mechanisms to resist not only endogenous ROS derived from the metabolic disturbance of bacteria during antibiotic treatment, but also exogenous ROS derived from the host immune system, are essential for bacterial survival (Cavinato, Genise, Luly, Di Domenico, Del Porto and Ascenzioni, 2020; Dryden, 2018). Usually, bacteria encode an antioxidant system to maintain oxidative balance within the bacterial cell. SOD, which is responsible for the reduction of superoxide, is one of the main components of this antioxidant system (Gao, Xia, Wang, Ye, Liu and Gao, 2019). *Salmonella enterica* encode three or more SOD proteins in their genomes. Studies on SODs in *S*. Typhimurium have focused solely on single-gene functions and yielded conflicting conclusions: SodCI, but not SodCII, contributes to virulence (Krishnakumar, et al., 2004), whereas SodCII has been reported to be associated with pathogenicity (Osman, et al., 2013); Fe/MnSOD (SodA) mediates serum resistance and biofilm formation (Wang, et al., 2018) yet has not been investigated in conjunction with other SOD family members. *S*. Pullorum, an important poultry pathogen, encodes four SODs. However, the roles and relative contributions of these individual SOD proteins to the stress resistance and virulence of *Salmonella* have remained unclear.

In our previous study, we found that knockout of (p)ppGpp synthetase (RelA and SpoT) caused reduced MBC and virulence of *S*. Pullorum (Wang, Cheng, Zhang, Lu, Wen, Luo, Shao, Pan and Zhang, 2021). (p)ppGpp synthetase is known to be responsible for synthesizing signaling molecules with regulatory effects, but not a direct effector (Zhang, et al., 2016). Through RNA sequencing analysis, the expression of *sod* genes was found to be reduced in the Δ*relA*Δ*spoT* mutant of *S.* Pullorum (Wang, Cheng, Zhang, Lu, Wen, Luo, Shao, Pan and Zhang, 2021). From this, we inferred that SOD proteins may be one of the important effectors of (p)ppGpp regulation, and thus may play a key role in antibiotic resistance and virulence of *S.* Pullorum. In this study, we further confirmed the importance of (p)ppGpp synthetase in antibiotic tolerance (Fig. 1). We then found that total SOD activity could be induced by antibiotic treatment, and this induction as well as the high expression of *sod* genes could be controlled by (p)ppGpp synthetase (Fig. 2). It has previously been reported that a series of regulators, such as PerR in Streptococci (Zhang, Ding, Li, Wan, Li, Chen and Zhou, 2012) and OxyR in *E. coli* (Sun, et al., 2022), participate in the regulation of antioxidant factors to balance ROS levels in bacterial cells. Even in some serious stresses, special regulatory pathways are needed to be against excessive ROS attacks, such as high expression of SOD in Methicillin-resistant *S. aureus* (MRSA) under protoporphyrin-mediated photodynamic inactivation (Nakonieczna, et al., 2010). Our results showed that the total SOD activity was (p)ppGpp synthetase-dependent during antibiotic treatment. This bears similarities to the regulation of SODs by (p)ppGpp synthetase in *P. aeruginosa*, which only has two SODs, both belonging to the Fe/MnSOD family (Nguyen, et al., 2011).

In order to comprehensively understand the influence of each of *S.* Pullorum’s four SOD proteins on bacterial resistance to antibiotics and host immune elimination, four *sod* single mutants (Δ*sod1*, Δ*sod2*, Δ*sod3,* and Δ*sod4*), and Cu/ZnSOD family (Δ*sod1*Δ*sod3*) and Fe/MnSOD family (Δ*sod2*Δ*sod4*) double mutants were constructed from the virulent *S*. Pullorum strain C79-3. We first compared the abilities of these *sod* mutants to resist superoxide stress and antibiotic treatment. Following paraquat treatment, the survival rates of all the single mutants were slightly reduced. Interestingly, the Cu/ZnSOD family double mutant Δ*sod1*Δ*sod3* had a similar slight reduction in survival, whereas the survival of the Fe/MnSOD family double mutant Δ*sod2*Δ*sod4* was highly reduced (Fig. 3B). The ability of the *sod*2 or *sod*4 mutants to resist antibiotic killing and tissue colonization was not significantly reduced compared with that of C79-3. Both Sod2 and Sod4 belong to the Fe/MnSOD family, we hypothesize that the loss of either protein may be functionally compensated by the other. The survival rates of these mutants under antibiotic treatment showed similar trends, with the Δ*sod2*Δ*sod4* mutant experiencing most significantly reduced antibiotic tolerance, resulting in swifter killing (Fig. 4). As an important component of the bacterial antioxidant system, SODs work to reduce excess and damaging ROS by degrading superoxide (Cavinato, Genise, Luly, Di Domenico, Del Porto and Ascenzioni, 2020). Given this, we then compared the ROS accumulation and total SOD activity of each strain during antibiotic treatment. As expected, the ciprofloxacin treatment caused a rapid increase in ROS. The Δ*sod2*Δ*sod4* mutant had the lowest total SOD activity and accumulated the highest amount of ROS, leading to the most serious ROS damage to proteins and lipids (Fig. 5, Fig. 6). It has previously been reported that accumulation of toxic ROS played a dominant role in rapid quinolone-mediated lethality of bacteria (Hong, et al., 2020). Our results further support the importance of antioxidation in antibiotic tolerance (Blázquez, et al., 2018), as total SOD activity was positively correlated with both, superoxide and antibiotic tolerance in *S*. Pullorum.

Since different families and numbers of SODs are encoded in the genomes of different species, the functional differences between SODs in different microorganisms can be difficult to tease apart. In *P. aeruginosa*, only two Fe/MnSODs are encoded, but no Cu/ZnSODs, and the Fe-cofactored SodB was found to be the dominant SOD, which played a crucial role in antibiotic tolerance in stationary phase (Martins, McKay, Sampathkumar, Khakimova, English and Nguyen, 2018). By contrast, a Cu/ZnSOD mutant of the fungal pathogen *Candida albicans* was more sensitive to menadione, a redox-cycling agent (Hwang, et al., 2002). In *S.* Pullorum, although all four SODs displayed some activity and contributed to superoxide tolerance, only the Δ*sod2*Δ*sod4* mutant had highly reduced superoxide and antibiotic tolerance. These results indicate that Fe/MnSODs (SOD2 and SOD4 collectively) play the major role in mediating antibiotic tolerance by countering ROS-derived bacterial damage. The dominance of Fe/MnSODs in *S*. Pullorum likely arises from adaptive evolution driven by its host microenvironment. First, the major infection sites of *S*. Pullorum (liver, spleen, and macrophages) are abundant in iron and manganese ions but deficient in copper and zinc (Gao et al., 2019; Zhou et al., 2023). Fe/MnSODs are better adapted to the ion availability in avian hosts. Second, under intense oxidative stress, Fe/MnSODs exhibit higher enzymatic stability and ROS‑scavenging efficiency than Cu/ZnSODs (Cavinato et al., 2020)

It is well known that, in addition to toxic ROS accumulation within bacteria during antibiotic killing, host immune cells also utilize ROS oxidative burst for pathogen elimination (de Jong, Parry, van der Poll and Wiersinga, 2012). Therefore, a pathogen’s ability to defend against oxidative damage is also particularly important for host-pathogen interactions during the context of infection. We have shown that *S.* Pullorum *sod* mutants have a reduced antioxidation ability, of which Δ*sod2*Δ*sod4* had the greatest loss of function. This might be the reason why the Δ*sod2*Δ*sod4* mutant had the lowest survival in macrophages and reduced colonization in chickens. However, it was still surprising that loss of Δ*sod2*Δ*sod4* resulted in a 100% survival rate (compared to 40% survival with the wild-type C79-3) and complete elimination of the bacteria by the host within a few days in both the spleen and liver, whereas the wild-type and all of the other mutants were able to persist (Fig. 7). These results suggest that the loss of the key antioxidant proteins SOD2 and SOD4 results in greatly attenuated pathogenicity in host animals. SODs have been recognized as a virulence factor in many pathogens (Gao, Xia, Wang, Ye, Liu and Gao, 2019). We think SODs appears to be because of their importance for promoting bacterial survival within the host rather than acting as direct virulence proteins that interacts with the host factors.

The presence of multiple SODs encoded in the genome raises questions regarding the contribution of each individual SOD to virulence. For example, in *S.* Typhimurium, there are four SODs. Wang et al. found that, Fe/MnSOD (SodA) was important in resistance to serum and oxidative stress, as well as helping to promote biofilm formation, adherence, and invasion of epithelial cells (Wang, Yi, Zhang, Sun, Wen, Zhang and Wang, 2018). Osman et al. found that Cu/ZnSOD (SodCⅡ) contributed to the virulence of *S*. Typhimurium (Osman, Patterson, Bailey, Fisher, Robinson, Rigby and Cavet, 2013), while Krishnakumar *et al.* suggested that another Cu/ZnSOD SodCⅠ, but not SodCⅡ, was crucial to the virulence of *S*. Typhimurium (Krishnakumar, Craig, Imlay and Slauch, 2004). These conflicting conclusions may arise from different parent strains and experimental conditions between these studies. In this study, we constructed these *sod* mutants in the same background (the virulent C79-3 strain) and experimental platform, and demonstrated that only the Fe/MnSODs double mutant (Δ*sod2*Δ*sod4*) was highly attenuated. Disentangling both the individual and cooperative contributions of each SOD will allow us to better understand their roles in the virulence of *S*. Pullorum.

Beyond impaired ROS scavenging, we infered that the attenuation of Δ*sod*2Δ*sod*4 may also involve disrupted intracellular metal cofactor homeostasis. As Fe/Mn-dependent metalloenzymes, Sod2 and Sod4 require available Fe/Mn ions for their activation and function. Deletion of *sod*2 and *sod*4 may disturb cellular Fe/Mn balance in *S*. Pullorum, impairing metal-dependent physiological processes related to stress resistance and virulence, and thus exacerbating attenuation independently of direct ROS damage.

It is interesting to note that only knockout of both Fe/MnSODs *sod2* and *sod4* resulted in the remarkable phenotypes of attenuated tolerance and virulence, but the attenuation of for each single mutant was very minor. A *sodA* or *sodB* single mutant of *E. coli* was also reportedly no more susceptible than WT to ampicillin, gentamicin, and norfloxacin killing (Wang and Zhao, 2009). Based on this, we can infer that these SOD proteins possess some the functional complementarity, and deletion of only one SOD within a family could not abolish its main functions. However, as reported in *P. aeruginosa*, Fe/MnSODs also have diverse functions and differential expression. *P. aeruginosa* SodB is the most abundant in iron-replete conditions, while SodA is under iron-dependent repression and only expressed under iron limitation (Hassett, Schweizer and Ohman, 1995). SodB was identified as the dominant SOD, which mediated antibiotic tolerance in stationary phase (Martins, McKay, Sampathkumar, Khakimova, English and Nguyen, 2018). By contrast, we only assessed bacteria cultured in LB broth in logarithmic phase. Although we did not test the expression of each *sod* in different nutrient conditions or growth phases, it appears that, at least under the conditions tested, both *sod2* and *sod4* were important with some functional redundancy, as only the Δ*sod2*Δ*sod4* had significantly attenuated antibiotic tolerance and pathogenicity.

## Conclusion

In conclusion, our results indicate that Fe/MnSODs (*sod2* and *sod4*) collectively have the most significant impact on antioxidation among the four SODs of *S*. Pullorum, and are essential for the antibiotic tolerance and virulence of this pathogen. This findings provide a theoretical basis for the prevention and control of pullorum disease based on antioxidant strategies.

## Funding

This work was supported by grants from the National Natural Science Foundation of China (32273014, 32503049), the National Key Research and Development Plan of China (2025YFD1800202) and the China Agriculture Research System (CARS-41).

## Ethics statement

All the animal experiments were approved (Permit number 11/2023) and supervised by the Institutional Animal Care and Use Committee of the Hubei Academy of Agriculture Sciences, Wuhan, China.

## CRediT authorship contribution statement

**Yiluo Cheng:** Writing – original draft, Resources, Methodology. **Yanli Li:** Software, Data curation. **Rhea Nickerson:** Formal analysis. **Wenting Zhang:** Software. **Yunqing Guo:** Software. **Qiao Hu:** Investigation. **Qin Lu:** Investigation. **Guoyuan Wen:** Visualization, Supervision. **Huabin Shao:** Conceptualization. **Zhenyu Cheng:** Writing – review & editing, Conceptualization. **Qingping Luo:** Project administration, Funding acquisition. **Tengfei Zhang:** Writing – review & editing, Project administration, Funding acquisition.

## Disclosures

The authors declare that they have no known competing financial interests or personal relationships that could have appeared to influence the work reported in this paper.

## Appendix Supplementary materials

Image, application 1

## Footnotes

Supplementary material associated with this article can be found, in the online version, at doi:10.1016/j.psj.2026.106638.
