# Supplementary material for: Iron-manganese superoxide dismutases are primarily responsible for antioxidative responses, antibiotic tolerance, and virulence of Salmonella Pullorum
Source: Poult Sci. 2026 Feb 13;105(5):106638. doi: 10.1016/j.psj.2026.106638 (PMC12926559; doi:10.1016/j.psj.2026.106638)
Supplement: Supplementary file 1 [file mmc1.docx]

Table S1. Primers designed and used in this study

| Primers by use | Primer sequences (5’ to 3’) | Targets |
| --- | --- | --- |
| Gene Knockout | | |
| R-sod1-F | TTATTATTTATCTTGCAAAAATATACCTGCTTTTATTAATGGTATTTACGATACAACCAAAAAACGAGGTAACTATGTGTAGGCTGGAGCTGCTTCG | *sod1* |
| R-sod1-R | GCCCTCCAGTGAGAGGGCTTTTTTATGGGTAAAACGAAATTATGACGATATGGCTATGTTGCTGCATATGAATATCCTCCTTAG |  |
| R-sod2-F | GCTCAATTTGCTACCCTATCCTTACGCGCACCAGGGGTATTGTGCGTGCGCAATATTAATAATAAGGAGAGAAGCATGTGTAGGCTGGAGCTGCTTCG | *sod2* |
| R-sod2-R | GGGCGTCTCCTCTATTCTATCCCTCTCTAAAACGCAGAAGGCGTACCTTCTGCGTACTCAGACCATATGAATATCCTCCTTAG |  |
| R-sod3-F | TGACTTTCCGTACATGTTTTCGCCAGTGGTTTACACTTAACAGGCGACCACATGTAACGGAGGTTTTTGTGTAGGCTGGAGCTGCTTCG | *sod3* |
| R-sod3-R | TCTGGCGTTTACACTGTTCGCTGTCGCAGCTGGAACAGGCTCCAGCGCAGGGAACACTGTCTCCGGGCATATGAATATCCTCCTTAG |  |
| R-sod4-F | TAACTATAATGAACCAACTGCTTACGCGGCGTTAACACTGTGCCGCTCGACAATAATGGAGATGATTTGTGTAGGCTGGAGCTGCTTCG | *sod4* |
| R-sod4-R | GCTGCTCCTTACGCGGATACAAAAAAAGCGAGTCATCAGACTCGCTTCTACAGTCGTGCAATGCAAACATATGAATATCCTCCTTAG |  |
| Gene complementation | | |
| C-sod1-F | CATGCATGCCACGTTATAGCGCGCTACCG | *sod1* |
| C-sod1-R | CGAGCTCTTATTTCTCAATGACACCACAGGCA |  |
| C-sod2-F | CATGCATGCTCATGAACCAGATTGGTAAACC | *sod2* |
| C-sod2-R | CGAGCTCTTATGCGGCGAGATTTTTC |  |
| C-sod3-F | CATGCATGCTTTTACGCCAGCGCAATTT | *sod3* |
| C-sod3-R | CGAGCTCTTATTTAATGACGCCGCAGG |  |
| C-sod4-F | CATGCATGCTATCGCCGCCCGTTTTCTCT | *sod4* |
| C-sod4-R | CGAGCTCTTATTTTTTAGCGGCGAAACG |  |
| qRT-PCR | | |
| sod1-qF | CTCCTCACCTAAATGGTCTTACG | *sod1* |
| sod1-qR | CGGAACCTCTTTACCGTCTTT |  |
| sod2-qF | GGATGGACATGGCTGGTAAA | *sod2* |
| sod2-qR | CGTTGCGGTAGTCGATGTAATA |  |
| sod3-qF | CTCCCGGTGTTAGTCGTTAAT | *sod3* |
| sod3-qR | GGATCATGAGCGCTTTATCTTTC |  |
| sod4-qF | TGCCGGTTGAAGAACTGATTA | *sod4* |
| sod4-qR | TTCCAGAAGAAGCTGTGGTTAG |  |
| gapdh-qF | GTTCTGACGGGTCCATCTAAAG | *gapdh* |
| gapdh-qR | GATGTCCTGGCCTTCGTATTT |  |

Table S2. Statistical analysis of bacterial survival rates under paraquat treatment

| significance analysis | | | P value |
| --- | --- | --- | --- |
| C79-3 | vs | Δsod1 | 0.00300000 |
|  |  | Δsod2 | 0.00000300 |
|  |  | Δsod3 | 0.00005400 |
|  |  | Δsod4 | 0.00100000 |
|  |  | Δsod1Δsod3 | 0.00010700 |
|  |  | Δsod2Δsod4 | 0.00000005 |
|  |  | ΔrelAΔspoT | 0.00000003 |
| Δsod1 | vs | C79-3 | 0.00300000 |
|  |  | Δsod2 | 0.00300000 |
|  |  | Δsod3 | 0.07700000 |
|  |  | Δsod4 | 0.64600000 |
|  |  | Δsod1Δsod3 | 0.14000000 |
|  |  | Δsod2Δsod4 | 0.00001700 |
|  |  | ΔrelAΔspoT | 0.00000900 |
| Δsod2 | vs | C79-3 | 0.00000300 |
|  |  | Δsod1 | 0.00300000 |
|  |  | Δsod3 | 0.11800000 |
|  |  | Δsod4 | 0.00700000 |
|  |  | Δsod1Δsod3 | 0.06400000 |
|  |  | Δsod2Δsod4 | 0.02300000 |
|  |  | ΔrelAΔspoT | 0.01200000 |
| Δsod3 | vs | C79-3 | 0.00005400 |
|  |  | Δsod1 | 0.07700000 |
|  |  | Δsod2 | 0.11800000 |
|  |  | Δsod4 | 0.17300000 |
|  |  | Δsod1Δsod3 | 0.73700000 |
|  |  | Δsod2Δsod4 | 0.00100000 |
|  |  | ΔrelAΔspoT | 0.00038000 |
| Δsod4 | vs | C79-3 | 0.00100000 |
|  |  | Δsod1 | 0.64600000 |
|  |  | Δsod2 | 0.00700000 |
|  |  | Δsod3 | 0.17300000 |
|  |  | Δsod1Δsod3 | 0.29400000 |
|  |  | Δsod2Δsod4 | 0.00004100 |
|  |  | ΔrelAΔspoT | 0.00002200 |
| Δsod1Δsod3 | vs | C79-3 | 0.00010700 |
|  |  | Δsod1 | 0.14000000 |
|  |  | Δsod2 | 0.06400000 |
|  |  | Δsod3 | 0.73700000 |
|  |  | Δsod4 | 0.29400000 |
|  |  | Δsod2Δsod4 | 0.00035800 |
|  |  | ΔrelAΔspoT | 0.00018900 |
| Δsod2Δsod4 | vs | C79-3 | 0.00000005 |
|  |  | Δsod1 | 0.00001700 |
|  |  | Δsod2 | 0.02300000 |
|  |  | Δsod3 | 0.00100000 |
|  |  | Δsod4 | 0.00004100 |
|  |  | Δsod1Δsod3 | 0.00035800 |
|  |  | ΔrelAΔspoT | 0.75900000 |
| ΔrelAΔspoT | vs | C79-3 | 0.00000003 |
|  |  | Δsod1 | 0.00000900 |
|  |  | Δsod2 | 0.01200000 |
|  |  | Δsod3 | 0.00038000 |
|  |  | Δsod4 | 0.00002200 |
|  |  | Δsod1Δsod3 | 0.00018900 |
|  |  | Δsod2Δsod4 | 0.75900000 |
